# TLR3 promotes MMP-9 production in primary human airway epithelial cells through Wnt/β-catenin signaling

**DOI:** 10.1186/s12931-017-0690-y

**Published:** 2017-12-13

**Authors:** P.-J. Royer, K. Henrio, M. Pain, J. Loy, A. Roux, A. Tissot, P. Lacoste, C. Pison, S. Brouard, A. Magnan, the COLT consortium

**Affiliations:** 1 UMR_S 1087 CNRS UMR_6291, l’Institut du Thorax, Université de Nantes, CHU de Nantes, Centre National de Référence Mucoviscidose Nantes-Roscoff, Nantes, France; 2 Hopital Foch, Pneumology, Adult Cystic Fibrosis Center and Lung Transplantation Department,Suresnes, France, Université Versailles Saint-Quentin-en-Yvelines, UPRESS EA220, Montigny le Bretonneux, Grenoble, France; 3 Clinique Universitaire de Pneumologie, Pôle Thorax et Vaisseaux, CHU Grenoble Alpes, Grenoble, France; 4 Université Grenoble Alpes, Grenoble, France; 5 Laboratoire de Bioénergétique Fondamentale et Appliquée, Inserm, 1055 Grenoble, France; 6 Centre de Recherche en Transplantation et Immunologie UMR1064, INSERM, Université de Nantes, Nantes, France; 7 Institut de Transplantation Urologie Néphrologie (ITUN), CHU Nantes, Nantes, France; 8 Faculté de Médecine, Université de Nantes, Nantes, France; 9 CIC Biotherapy, CHU Nantes, Nantes, France

## Abstract

**Background:**

Airway epithelial cells (AEC) act as the first line of defence in case of lung infections. They constitute a physical barrier against pathogens and they participate in the initiation of the immune response. Yet, the modalities of pathogen recognition by AEC and the consequences on the epithelial barrier remain poorly documented.

**Method:**

We investigated the response of primary human AEC to viral (polyinosinic-polycytidylic acid, poly(I:C)) and bacterial (lipopolysaccharide, LPS) stimulations in combination with the lung remodeling factor Transforming Growth Factor-β (TGF-β).

**Results:**

We showed a strong production of pro-inflammatory cytokines (Interleukin (IL)-6, Tumor Necrosis Factor α, TNFα) or chemokines (CCL2, CCL3, CCL4, CXCL10, CXCL11) by AEC stimulated with poly(I:C). Cytokine and chemokine production, except CXCL10, was Toll Like Receptor (TLR)-3 dependent and although they express TLR4, we found no cytokine production after LPS stimulation. Poly(I:C), but not LPS, synergised with TGF-β for the production of matrix metalloproteinase-9 (MMP-9) and fibronectin. Mechanistic analyses suggest the secretion of Wnt ligands by AEC along with a degradation of the cellular junctions after poly(I:C) exposure, leading to the release of β-catenin from the cell membrane and stimulation of the Wnt/β-catenin pathway.

**Conclusion:**

Our results highlight the cross talk between TGF-β and TLR signaling in bronchial epithelium and its impact on the remodeling process.

**Electronic supplementary material:**

The online version of this article (10.1186/s12931-017-0690-y) contains supplementary material, which is available to authorized users.

## Background

Airway epithelial cells (AEC) act as a physical barrier, separating the inside of the body from the environment through a dense network of transcellular adherens and tight junctions. Disorganisation of airway epithelium is a hallmark of chronic respiratory disorders. It leads to airway obstruction and loss of respiratory function. Airway remodeling through the process of epithelial to mesenchymal transition (EMT) has been proposed in chronic respiratory diseases such as chronic obstructive pulmonary disease (COPD), pulmonary fibrosis, asthma or chronic lung allograft dysfunction [1–3]. However, airway epithelium has more than a structural role. It is the first line of defence against airborne pathogens, particulate matter or allergens and express a wide range of pathogen recognition receptors such as Toll Like Receptor (TLR), the Nucleotide Oligomerization Domain (NOD)-like receptors (NLR) or the Retinoic acid-Inducible Gene 1 (RIG-I)-like receptor (RLR). Recent works have highlighted the capacity of AEC to attract and activate innate or adaptive immune cells within the lung. AEC are thus the main orchestrators of the lung inflammatory responses and remodeling processes associated to the respiratory physiology [4, 5].

Relationship between remodeling and inflammation has been suggested for a long time [6]. Matrix metalloproteinases (MMP) produced during mesenchymal differentiation spread inflammation through the degradation of matrix and lung tissues, or through the processing of cytokines and chemokines allowing the recruitment of immune cells within the lung [7–9]. Transforming growth factor β (TGF-β) is the foremost inducer of EMT. It plays a central role in the pathological mechanisms behind the remodeling process observed in COPD, pulmonary fibrosis or asthma [10–12]. However, TGF-β is a pleiotropic growth factor expressed in the normal lung [13], and its ability to drive EMT is largely dependent on the microenvironment in which it is produced. Inflammatory cytokines like TNFα and IL-1β [14, 15], or chemokines like CCL-2 [16] produced by macrophages or activated T cell have thus been shown to promote TGF-β-induced EMT.

Although AEC express a wide range of pathogen recognition receptors, their contribution on TGF-β-induced EMT remains largely unexplored. We investigated here the impact of pathogens associated molecular patterns (PAMPS) on AEC remodeling during TGF-β exposure. Primary human AEC were cultured under submerged conditions or were differentiated at Air Liquid Interface (ALI). Viral (polyinosinic-polycytidylic acid (poly(I:C)) or bacterial (lipopolysaccharide (LPS)) stimulations were used along with low-dose of TGF-β (1 ng/ml). The inflammatory response as well as the remodeling process engaged were assessed. We showed that poly(I:C), but not LPS, synergised with TGF-β for the production of matrix metalloproteinase-9 (MMP-9) and fibronectin. Mechanistic analyses suggest the secretion of Wnt ligands by AEC along with a degradation of the cellular junctions after poly(I:C) exposure, leading to the release of β-catenin from the cell membrane and stimulation of the canonical Wnt/β-catenin pathway. Our results highlight the cross talk between TGF-β, TLR and Wnt signaling in bronchial epithelium and its impact on the remodeling process.

## Methods

### Airway epithelial cell culture

AEC were isolated and cultured as already described [16]. Briefly, human primary BEC were obtained from lung donor trachea or bronchi, included within the multicentre COLT (Cohort in Lung Transplantation, NCT00980967) study (Comité de Protection des Personnes Ouest 1-Tours, 2009-A00036–51). Study was approved by local ethical committee. After excision, tissues were incubated at 4 °C overnight with 1 mg/ml type XIV collagenase in HEPES-buffered RPMI (both from Sigma-Aldrich). Isolated AEC were washed and cultured for less than 5 passages in cnT17 (CELLnTEC Advanced Cell Systems AG, Bern, Switzerland) containing penicillin and streptomycin (P/S) (respectively 100 UI/ml and 100 μg/ml) on 24-well plates coated with human type IV collagen (Sigma-Aldrich). Cell purity was routinely checked by cytokeratin and αSMA staining. For air-liquid interface (ALI) cultures, cells were grown at confluence in cnT17 medium, on 12-mm diameter, 0.4-μm pore size transwells (Corning, NY) coated with human type IV collagen. Then, 1 ml of a 1:1 mix of DMEM (Gibco, Invitrogen Ltd., Paisley, UK) and Bronchial Epithelial Basal Medium + bullet kit (Lonza, Basel, Switzerland) (Bronchial Epithelial Growth Medium, BEGM) supplemented with retinoic acid (1.10^−7^ M), bovine serum albumin (1.5 μg/ml) and P/S (all from Sigma-Aldrich) was added to the basal compartment. Medium was changed every 2–3 days and ALI cultures were used after 21 days. ALI differentiation was verified by β-tubulin/β-catenin staining.

Submerged or ALI-differentiated AEC were then stimulated in CnT17 or BEGM respectively, supplemented with 0.5% foetal calf serum (Sigma-Aldrich). AEC were treated with 1 ng/ml of TGF-β (R&D systems, Abingdon, UK), 1 μg/ml of LPS or 50 μg/ml of poly(I:C) (both from Sigma-Aldrich). Activity of TLR ligands was verified on human PBMC (not shown). For ALI-culture, TGF-β was added at the lower compartment and LPS (1 μg in 20 μl) and poly(I:C) (50 μg in 20 μl) were added to the apical surface of the epithelium. In some conditions, AEC were treated with 100 μM of 614,310 (inhibitor of TLR3/dsRNA complex) (Calbiochem, Merck Millipore, Fontenay Sous Bois, France), 5 μM of CID755673 (inhibitor of PKD), 1 μM of FH535 (inhibitor of the β-catenin/ T-cell factor/lymphoid enhancer-binding factor (TCF/LEF)) (both from Tocris, Bio-techne, Lille France) and 5 μM of IWP2 (inhibitor of Wnt ligand secretion) (Sigma Aldrich). Inhibitors were added 30 min. Before AEC stimulation and were kept in the medium during the culture. After 24 h of culture, supernatants were collected, centrifuged for 5 min at 13,000 g and frozen at −20 °C for subsequent analysis. AEC were rinsed with PBS (Gibco) before RNA and protein extraction.

### RT^2^ profiler PCR array and quantitative PCR

RNA were extracted using the RNA NucleoSpin kit (Macherey-Nagel, Hoerdt, France). The human EMT RT^2^ profiler PCR array (Qiagen) was utilized to investigate a panel of 84 EMT related genes according to the manufacturer’s instructions. Briefly, 500 ng of RNA was converted to cDNA using RT^2^ First Strand Kit (Qiagen). cDNA was amplified by qPCR in RT^2^ SYBR Green qPCR Master Mix (Qiagen), using a Bio-rad CFX96 system. Analysis of expression was then performed using the web-based RT^2^ profiler PCR Array data analysis software (Qiagen). PCR array results were then confirmed by qPCR using SYBR green mix (Eurogentec, Angers, France) on a CFX96 system using the following primers: GAPDH forward 5′-GGGAAACTGTGGCGTGAT-3′, reverse 5′-TTCAGCTCAGGGATGACCTT-3′; MMP-9 forward 5′-ATGCCTGCAACGTGAACATCTTCG-3′, reverse 5′-CAGAGAATCGCCAGTACTTCCCAT-3′; Fibronectin forward 5′-GCAGGGTCAGCAAATGGTTCAG-3′, reverse 5′-AGGTAGGTCCGCTCCCACTG-3′; ColVA2 forward 5′-ATGGTCCTGATGGCCCAAAA-3′, reverse 5′-AATTCCTCTTTCTCCCGGCA-3′; ITGA5 forward 5′-CTTCCTCGGGACCTCAGATC-3′, reverse 5′-TTTGGCTCTCTTGTTGGTGC-3′; PLEK2 forward 5′-GTATGAAAACCGACCGCTCC-3′, reverse 5′-GAATAGCCCCGGTGATCTCA-3′, BAMBI forward 5′-GATGCTACTGTGATGCTGCC-3′, reverse 5′-TGGGTGAGTGGGGAATTTGA-3′; Wnt3a forward 5′-AGAGGCGGGGCTACAGATT-3′, reverse 5′-CAGAGCCACGCCCTTACTG-3′; Wnt4 forward 5′-GCAGAGCCCTCATGAACCT-3′, Wnt4 reverse 5′-CACCCGCATGTGTGTCAG-3′; Wnt5a forward 5′-CGTCTGGAAGCAGACGTTTC-3′, reverse 5′-TCACGCCTCCTGATCTCC-3′; Wnt5b forward 5′-TGCCTTTCCAGCGAGAAT-3′, Wnt5b reverse 5′-CCCCCTTTCCTCTTCAGGTA-3′; Wnt7a forward 5′-CTTCGGGAAGGAGCTCAAA-3′, reverse 5′-GCAATGATGGCGTAGGTGA-3′; Wnt10a forward 5′-TAATTCCATAAAGGGCCCAGA-3′, reverse 5′-TTGTTAAATGAATGATGAAGG-3′. Gene expression was normalized to GAPDH.

### Immunoblotting

Cellular fractionation was performed with the nuclear protein extraction kit (Cayman Chemical, Hamburg, Germany) according to the manufacturers’ instructions. Proteins extracted with the RNA NucleoSpin kit separated on 8% bis-acrylamide gel were transferred onto nitrocellulose membranes (Bio-Rad). Membranes were blocked for 1 h at room temperature in Tris-buffered saline (TBS) (100 mM NaCl, 10 mM Tris, pH 7.5) with 5% non-fat dry milk. Membranes were incubated overnight at 4 °C with primary antibodies against the following proteins: fibronectin (Santa Cruz, Heidelberg, Germany), β-actin (Sigma-Aldrich), active (#8814) and total (#8480) β-catenin (both from Cell Signaling, Leiden, The Netherlands). Detection was performed with horseradish peroxydase-labelled secondary antibodies (Rockland Immunochemicals, Limerick, USA) and a clarity chemiluminescence kit (Bio-Rad). Chemidoc MP imaging (Bio-Rad) was used for analysis.

### Immunofluorescence

β-catenin and E-cadherin staining were performed on methanol fixed submerged cultures. Primary antibody against total β-catenin (Cell Signaling) and E-cadherin (Invitrogen) were used followed by an Alexa Fluor 568-conjugated anti-rabbit IgG or an Alexa Fluor 488-conjugated anti-mouse IgG (both from Life Technologies). Nuclei were stained with DAPI and samples were mounted with Prolong Gold Antifade reagent (Life Technologies). Images were taken using Axiovert 200 M microscope and analyzed with Axiovision 4.5 software (Carl Zeiss; Marly Le Roi, France).

### Detection of cytokines and MMP-9

Cytokine concentrations were determined with the FlowCytomix technology and levels of MMP-9 in cell culture supernatants were measured with the human MMP-9 platinum ELISA (both from Thermofisher, Villebon sur Yvette, France).

### Statistical analysis

Data are expressed as mean +/− standard error of mean (SEM). Number of independent repeats is stated in the figure legends. Differences between groups were assessed by one-way analysis of variance (ANOVA) followed by a Tukey post hoc test using GraphPad Prism 6.0 (GraphPad Software; La Jolla, USA). * *p* < 0.05, ** *p* < 0.01, *** *p* < 0.001.

## Results

### Proinflammatory response of AEC after stimulation with TLR agonists

**Fig. 1:**
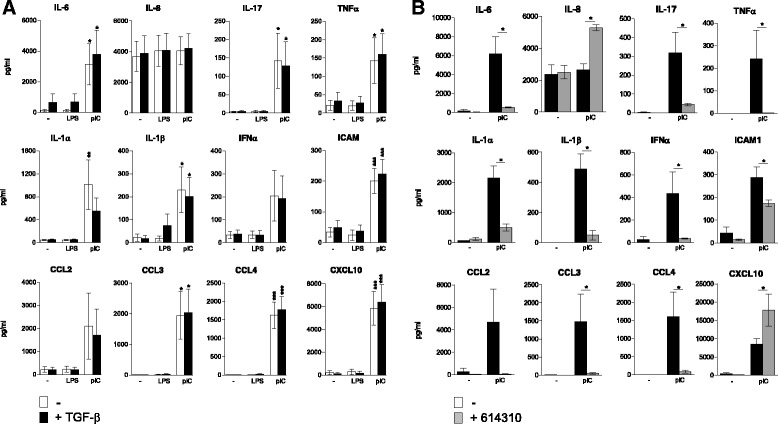
Inflammatory response of airway epithelial cells exposed to poly(I:C) or LPS. **a** Primary human AEC were cultured under submerged conditions without (−) or with poly(I:C) (pIC) or LPS (LPS) in presence or not of TGF-β.Cytokine and chemokine secretion was investigated after 24 h of culture. **b** AEC were pretreated with a TLR3/dsRNA complex inhibitor (614310) before analysis of cytokine and chemokine secretion. Data in A and B are derived from 6 and 3 independent experiments. Statistical significances were determined with a one-way ANOVA followed by a Tukey’s post-hoc test

We first investigated the inflammatory response of AEC after stimulation with LPS or poly(I:C), a double stranded RNA, to simulate bacterial or viral interaction respectively. We showed a strong production of inflammatory cytokines (IL-6, IFNα, IL-1α and β), chemokines (CCL2, 3, 4 and CXCL10) and soluble ICAM-1 immunoglobulin after treatment with poly(I:C) but not LPS (Fig. 1a). Moderate levels TNF-α and IL-17 were produced. IL-8 production by contrast was not affected by the culture conditions. This inflammatory response initiated by poly(I:C) exposure was not altered by TGF-β. Whereas TLR3 is the main receptor for double-stranded RNA, Retinoid induce gene I (RIG-1) and Melanoma Differentiation-Associated protein 5 (MDA5), two cytosolic RLR have been described as well [17]. To confirm the role of TLR3 in dsRNA-induced inflammatory response, AEC were treated with a TLR3/dsRNA complex inhibitor (614310) before poly(I:C) stimulation. We noted a dramatic drop in cytokine (IL-6, TNFα, IL-17, IL-1α and β) and chemokine (CCL2, CCL3 and CCL4) secretion after TLR3 blockade (Fig. 1b) although ICAM1 production was partially affected. Surprisingly, IL-8 and CXCL10 production was upregulated, suggesting a RLR dependent production and a cross interference between the RLR and TLR signaling.

### Poly(I:C) supports TGF-β induced EMT process in human AEC

**Fig. 2:**
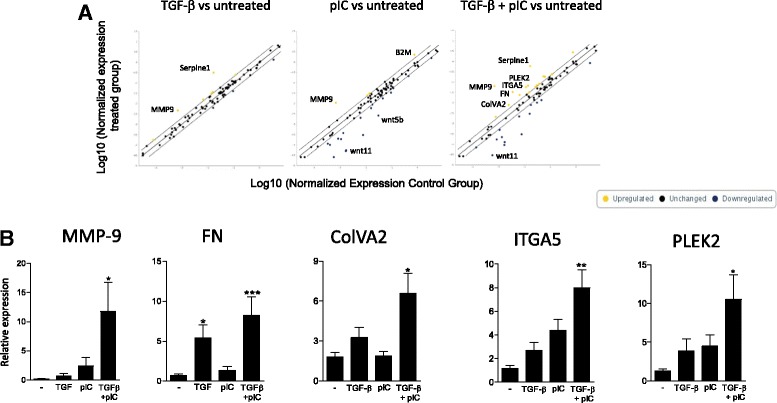
Poly(IC) support epithelial to mesenchymal transition in AEC treated with TGF-ß. Human primary AEC were cultured under submerged conditions with TGF-β and/or poly(I:C) for 24 h. **a** Expression of EMT related genes was investigated using a profiler PCR array (*n* = 1). **b** qPCR from 3 independent experiments confirmed the expression data. Statistical significances were determined with a one-way ANOVA followed by a Tukey’s post-hoc test

We then investigated the ability of a TLR stimulation to support the process of EMT in AEC. Pathway focused gene expression analysis using an EMT profiler array was performed to screen AEC response to TGF-β, poly(I:C) or the combination of the two. Untreated AEC were used as a control. We noted an upregulation of MMP-9 (fold change (FC) = +6.6) and serpin (FC = +18.6) after TGF-β treatment, while poly(I:C) alone notably induced MMP-9 upregulation (FC = +15.2) and downregulation of Wnt5b (FC = −12.1) and Wnt11 (FC = −34.9). Combination of TGF-β and poly(I:C) promoted a strong upregulation of MMP-9 (FC = +90.9), serpine1 (FC = +34.8), fibronectin (FC = +9.2), Collagen type V, alpha2 (ColVA2) (FC = +3.1), Integrin A5 (ITGA5) (FC = + 4.9) or Pleckstrin 2 (PLEK2) (FC = +4.8) along with a downregulation of Wnt11 (FC = −19.1) (Fig. 2a and Additional file 1: Table S1). We then performed independent cell cultures to confirm by qPCR the EMT array data. Profile of expression MMP-9, fibronectin, ColVA2, ITGA5 and PLEK2 was notably confirmed, with a dramatic and significant upregulation of expression in the TGF-β + poly(I:C) condition (Fig. 2b).

### Production of MMP-9 by poly(I:C) is TLR3 dependent but is not related to a stimulation of TGF-β production or signaling

**Fig. 3:**
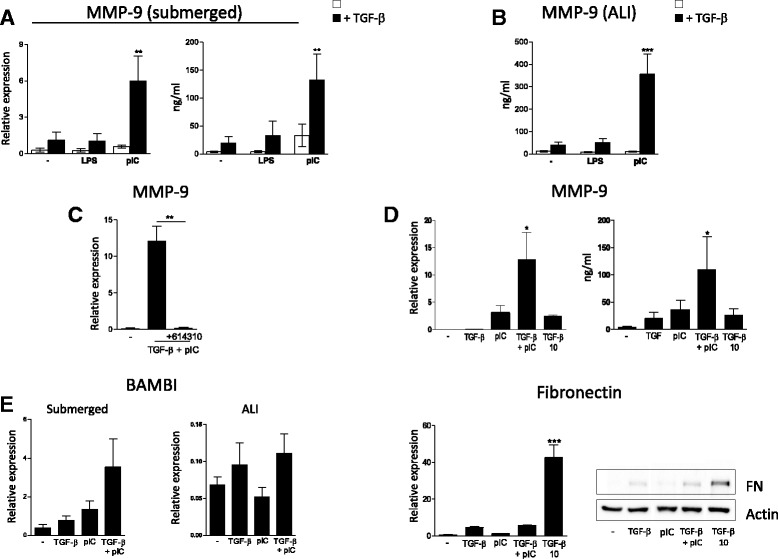
Analysis of MMP-9 production by airway epithelial cells exposed to poly(I:C) and TGF-β. **a** MMP-9 production was investigated in submerged cultures by qPCR or ELISA dosage (*n* = 4). **b** Results of expression were then confirmed in ALI culture conditions by ELISA (*n* = 6). **c** Use of TLR3/dsRNA complex inhibitor (614310) shows the role of TLR3 in MMP-9 production (*n* = 3). **d** Primary human AEC were cultured for 24 h with increasing doses of TGF-β, and MMP-9 production was measured by qPCR or ELISA dosage (n = 3). Fibronectin expression was investigated by qPCR (n = 3) or western Blotting. **e** qPCR analysis of BAMBI expression in submerged or ALI cultures exposed to TGF-β and/or poly(I:C) for 24 h (n = 3). Number of independent experiments is mentioned for each panel. Statistical significances were determined with a one-way ANOVA followed by a Tukey’s post-hoc test

We then sought to explore the molecular mechanisms behind the synergistic interaction between TGF-β and poly(I:C) by monitoring the expression of MMP-9, the gene with the highest FC. qPCR and dosage from submerged cultures showed that poly(I:C) but not LPS supported TGF-β-induced MMP-9 production by AEC (Fig. 3a). These results were confirmed in ALI culture conditions (Fig. 3b). Then, experiments with the TLR3/dsRNA complex inhibitor confirmed the role of TLR3 signaling in the interaction between TGF-β and poly(I:C) (Fig. 3c).

The upregulation of MMP-9 was not a consequence of TGF-β overproduction after poly(I:C) treatment as we could not detect any secretion of the latency associated peptide (not shown), generated during mature TGF-β release. Moreover, MMP-9 production under high TGF-β concentration remained lower than with the TGF-β + poly(I:C) combination (Fig. 3d), although this is not the case for fibronectin expression. Besides, qPCR experiments did not reveal any downregulation of BMP and activin membrane-bound inhibitor homolog (BAMBI) expression, a TGF-β pseudo receptor known to inhibit TGF-β signaling [18] (Fig. 3e). Altogether, these results ruled out a raise of TGF-β production or signaling as the origin of MMP-9 release.

### Poly(I:C) supports TGF-β induced MMP-9 through Wnt/β-catenin dependent mechanism

**Fig. 4:**
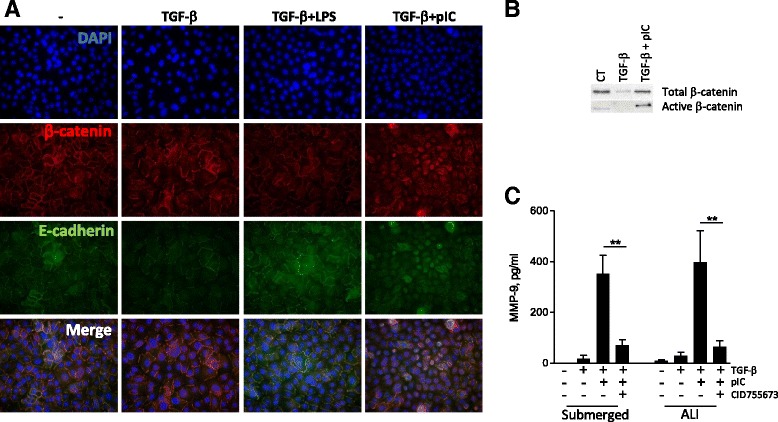
Relocation of β-catenin after TGF-β and poly IC treatment. **a** Immunofluorescence analysis of β-catenin and E-cadherin expression in submerged human AEC exposed to TGF-β and/or poly(I:C) (×40). **b** After cellular fractionation, nuclear levels of active and total β-catenin was investigated by western blotting. **c** Primary AEC cultured under submerged or ALI conditions were treated with an inhibitor of PKD (CID755673) before stimulation for 24 h with TGF-β and/or poly(I:C). Levels of MMP-9 were then determined by ELISA. Data are derived from 3 independent experiments and statistical significances were determined with a one-way ANOVA followed by a Tukey’s post-hoc test

**Fig. 5:**
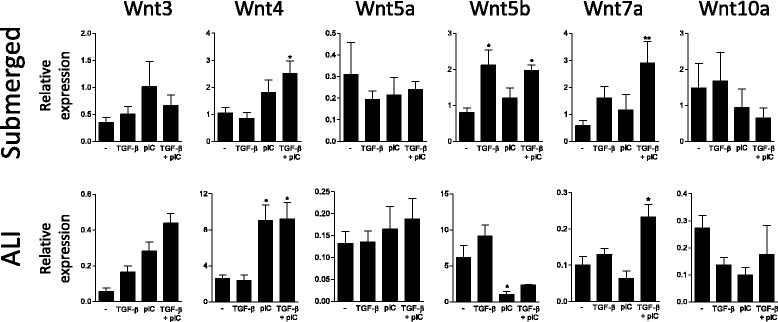
Analysis of Wnt expression by human primary AEC. qPCR analysis of Wnt ligand expression in primary AEC cultured under submerged or ALI conditions with TGF-β and/or poly(I:C). Data are derived from 4 independent experiments and statistical significances were determined with a one-way ANOVA followed by a Tukey’s post-hoc test

**Fig. 6:**
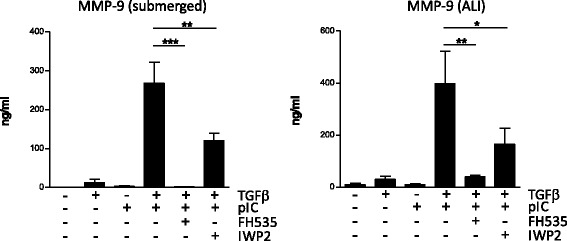
Inhibition of Wnt/β-catenin signaling blocks MMP-9 production. Human primary AEC cultured under submerged or ALI conditions were treated with FH535 or IWP2 to block respectively the β-catenin/TCF/LEF complex or Wnt secretion. AEC were then stimulated with TGF-β and/or poly(I:C) for 24 h before analysis of MMP-9 release by ELISA. Data are derived from 4 independent experiments and statistical significances were determined with a one-way ANOVA followed by a Tukey’s post-hoc test

MMP-9 is a known target of Wnt/β-catenin signaling [19]. Because TLR3 can induce the disruption of epithelial barrier [20], we hypothesized that TLR3 signaling induces the release of β-catenin from the adherens junction, and the subsequent production of MMP-9. Epithelial junctions in AEC were then investigated by fluorescent microscopy. Treatment with TGF-β or TGF-β + LPS did not alter the E-cadherin and β-catenin membrane staining (Fig. 4a). By contrast, we noted a loss of membrane E-cadherin and β-catenin after TGF-β + poly(I:C) treatment, attesting a disassembly of adherens junctions, AEC appear then smaller when cultured in TGF-β and poly(I:C). The transfer of the active form of β-catenin in the nucleus was confirmed by subcellular fractionation (Fig. 4b). Inhibition of protein kinase D (PKD), known to mediate the disruption of epithelial barrier after poly(I:C) exposure [20], reduced MMP-9 secretion in submerged or ALI differentiated AEC (Fig. 4b). We then determined by qPCR the expression of Wnt ligands from submerged or ALI cultures. The expression of the canonical (β-catenin dependent) Wnt7a was upregulated in submerged or ALI cultures after TGF-β + poly(I:C) stimulation (Fig. 5). Although Wnt4 and Wnt5a have been primarily described as non-canonical Wnt, recent findings show that they can activate the β-catenin pathway under certain conditions [21]. We thus investigated their expression and showed an upregulation of Wnt4 expression in submerged or ALI condition after TGF-β + poly(I:C) treatment. Wnt 2 however was not detected (not shown). Finally, to further confirm the role of β-catenin signaling in MMP-9 production, submerged or ALI cultures of AEC were performed in presence of the following inhibitors: FH535, an inhibitor of the β-catenin/TCF/LEF activity, and IWP2, an inhibitor of Wnt ligand secretion. Both inhibitors induced a dramatic drop in MMP-9 secretion (Fig. 6).

## Discussion

AEC occupy a central position at the interface between environment and the body. They are continuously exposed to the external stimuli and regulatory mechanisms have been developed to avoid a permanent hyper activation of the immune system. Whereas they are equipped with a wide range of TLR, they express low levels of adapter proteins such as MD2 or CD14 reducing their responsiveness to bacteria-derived signals [22]. Secretion of IL-6 or IL-8 has been reported, but with high dose of LPS (≥10 μg/ml) and the used of cell lines rather than primary cells [23–25]. The absence of response of AEC to LPS is thus expected. Viral stimulations by contrast induce a potent pro-inflammatory response as shown by IL-6, IL-8 and type I IFN production [24]. We confirmed here the induction of inflammatory response by primary human AEC after viral-derived stimulation. We used double-stranded RNA as it is a product of most virus infections (by either DNA or single-stranded RNA viruses) [26]. Double stranded RNA can be recognized by TLR3, but also the cytosolic MDA5 or RIG-1 RLR. We showed here TLR3 as the main actor of the inflammatory response in AEC. Interestingly, IL-8 and CXCL10 secretion profiles suggested their production in a NLR dependent mechanism, and a cross interference between TLR and NLR pathways. This interplay between pathogen recognition receptors has been reported before [27]. However, further work will be necessary to determine the contribution of NLR in AEC inflammatory response and to confirm in these cells the cross interference between RLR and TLR pathways.

Recent literature highlighted the major role of AEC in the remodeling processes associated with chronic diseases, infections or transplantation [1]. Although modulation of TGF-β induced remodeling by inflammatory environment has been described [14–16], the direct interaction between TGF-β and TLR signaling in AEC remains unexplored. AEC were cultured with low dose of TGF-β (1 ng/ml) to emphasize the synergy with TLR3 signaling. Using profiler array, we showed that poly IC supports TGF-β induced EMT with a significant upregulation of *MMP-9*, *FN*, *COLVA1*, *ITGA5* or *PLEK2* expression. Interestingly, we recently described how T cells-derived signals promote MMP-9 or FN production by AEC exposed to TGF-β [16]. These results collectively suggest that in response to various aggression/stimulation signals, AEC generate a common remodeling signature.

**Fig. 7:**
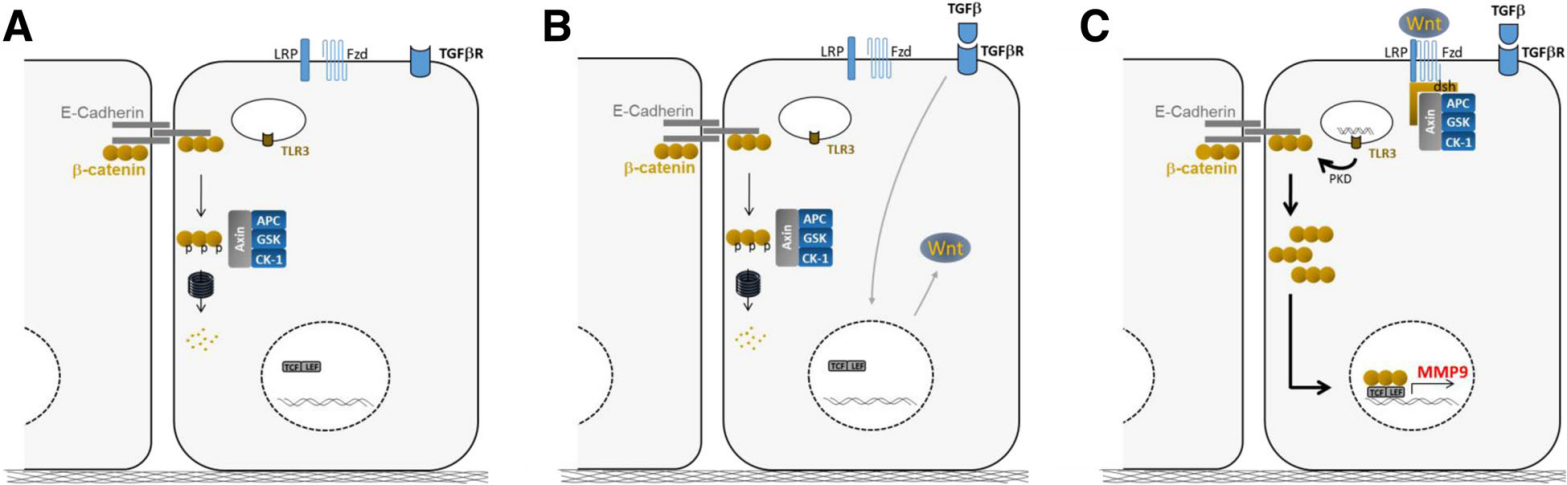
Summary of the main findings. **a** Steady-state: cytosolic β-catenin is phosphorylated by the GSK complex and targeted to the proteasome for degradation. **b** Wnt ligand production after TGF-β exposure stabilize GSK complex at the cell membrane and reduce β-catenin degradation. **c** Then, the massive relocation of β-catenin after poly(I:C) treatment, fuels the Wnt/β-catenin pathway and allows β-catenin translocation in the nucleus for MMP-9 expression

We further investigated the molecular mechanisms leading to MMP-9 production when TGF-β and poly(I:C) were combined. Indeed, the role of MMP-9 is not limited to tissue remodeling. It can propagate inflammatory response through the modulation of cytokine activity and recruitment of immune cells [28]. Its production along with inflammatory cytokines, provide immune cells with chemoattractant, penetrable extracellular matrix, and activation signals. We did not detect any production of TGF-β by AEC and increasing TGF-β concentration did not raise MMP-9 secretion. Moreover, TGF-β signaling appeared unaffected in our model. Thus, TGF-β production or gain in signaling does not account for the production of MMP-9. Wnt/β-catenin signaling is essential during lung development, whereas aberrant Wnt/β-catenin is involved in the remodeling processes associated with chronic lung diseases [1, 29]. Growing evidences show environmental factors such as cigarette smoke, as inducers of Wnt ligands in AEC [30, 31]. In an infectious context, Rezaee et al. reported a PKD-dependent cytosolic transfer of β-catenin in AEC treated with poly(I:C) but not LPS [20]. Yet, the authors focused on the organization of the apical junctions, and further investigations of Wnt/β-catenin signaling were not performed. Given the massive production of MMP-9, a target of Wnt/β-catenin signaling [19], we hypothesized that this pathway was engaged in our model. We showed the production of Wnt ligands by AEC after exposure to TGF-β or poly(I:C). Wnt ligands are a family of lipid-modified cysteine-rich secreted proteins. Nineteen Wnt ligands have been identified so far [32]. Although we focused on lung relevant ones, other Wnt ligands not investigated in our study could be mobilized as well. Moreover, cellular context can favor either the canonical or the non-canonical pathway [33]. Additional work will thus be necessary to confirm *Wnt gene* expression and for a comprehensive overview of the Wnt production by AEC, and for determining which particular ligand(s) is (are) involved in our model. The release of β-catenin from cell membrane after poly(I:C) stimulation fuels the Wnt/β-catenin pathway. The transfer of β-catenin to the nucleus then coincides with the massive production of MMP-9 in a PKD dependent manner. A positive feedback loop, involving the degradation of cell-cell junctions by MMP-9 is possible but was not investigated in our study. Using inhibitors of Wnt secretion or β-catenin/TCF/LEF activity we finally confirmed the role of Wnt/β-catenin pathway in the production of MMP-9. A summary of our main findings, is presented in Fig. 7.

Our study has several limitations that need to be pointed out. Our experimentations were exclusively performed with PAMPS and our findings will have to be confirmed with viable pathogens. Moreover, MMP-9 is produced by different cell types like neutrophils or macrophages, and the relative contribution of these cells to lung MMP-9 production remains to be explored.

## Conclusion

As a conclusion, we describe how the cross talk between TGF-β and TLR3 signaling favours a remodeling process in the bronchial epithelium. Our work supports the airway epithelium as an initiator of immune responses and identifies the Wnt/β-catenin pathway as a potential therapeutic target to tackle the progression of the remodeling process associated to respiratory diseases.

## Additional file

Additional file 1: Table S1. Expression data from the EMT profiler array. (XLSX 15.9 kb)

## Abbreviations

AEC: Airway Epithelial Cells
ALI: Air Liquid Interface
BAMBI: BMP and Activin Membrane-Bound Inhibitor homolog
COPD: Chronic Obstructive Pulmonary Disease
EMT: Epithelial to Mesenchymal Transition
LPS: Lipopolysaccharide
MDA5: Melanoma Differentiation-Associated protein 5
MMP: Matrix MetalloProteinase
NLR: Nucleotide Oligomerization Domain (NOD)-Like Receptors
PAMP: Pathogen Associated Molecular Pattern;
PKD: Protein Kinase D
poly(I:C): polyinosinic-polycytidylic acid
RIG-1: Retinoic acid-Inducible Gene I
RLR: RIG-I-like receptor
TCF/LEF: T-Cell Factor/Lymphoid Enhancer-binding Factor
TGF-β: Transforming Growth Factor β
TLR: Toll Like Receptor
TNF-α: Tumor Necrosis Factor α

## References

[1] Pain M, Bermudez O, Lacoste P, Royer P-J, Botturi K, Tissot A, Brouard S, Eickelberg O, Magnan A (2014). Tissue remodelling in chronic bronchial diseases: from the epithelial to mesenchymal phenotype. Eur Respir Rev.

[2] Royer P-J, Olivera-Botello G, Koutsokera A, Aubert J-D, Bernasconi E, Tissot A, Pison C, Nicod L, Boissel J-P, Magnan A, Sys Cc (2016). Chronic lung allograft dysfunction: a systems review of mechanisms. Transplantation.

[3] Kage H, Borok Z (2012). EMT and interstitial lung disease: a mysterious relationship. Curr Opin Pulm Med.

[4] Weitnauer M, Mijošek V, Dalpke AH (2016). Control of local immunity by airway epithelial cells. Mucosal Immunol.

[5] Whitsett JA, Alenghat T (2015). Respiratory epithelial cells orchestrate pulmonary innate immunity. Nat Immunol.

[6] Hirota N, Martin JG (2013). Mechanisms of airway remodeling. Chest.

[7] Bradley LM, Douglass MF, Chatterjee D, Akira S, Baaten BJG (2012). Matrix metalloprotease 9 mediates neutrophil migration into the airways in response to influenza virus-induced toll-like receptor signaling. PLoS Pathog.

[8] Atkinson JJ, Senior RM (2003). Matrix metalloproteinase-9 in lung remodeling. Am J Respir Cell Mol Biol.

[9] Houghton AM (2015). Matrix metalloproteinases in destructive lung disease. Matrix Biol.

[10] Shi W, Chen F, Cardoso WV (2009). Mechanisms of lung development. Proc Am Thorac Soc.

[11] Halwani R, Al-Muhsen S, Al-Jahdali H, Hamid Q (2011). Role of transforming growth factor-β in airway remodeling in asthma. Am J Respir Cell Mol Biol.

[12] Fernandez IE, Eickelberg O (2012). The impact of TGF-β on lung fibrosis: from targeting to biomarkers. Proc Am Thorac Soc.

[13] Magnan A, Frachon I, Rain B, Peuchmaur M, Monti G, Lenot B, Fattal M, Simonneau G, Galanaud P, Emilie D (1994). Transforming growth factor beta in normal human lung: preferential location in bronchial epithelial cells. Thorax.

[14] Borthwick LA, Corris PA, Mahida R, Walker A, Gardner A, Suwara M, Johnson GE, Moisey EJ, Brodlie M, Ward C (2013). TNFα from classically activated macrophages accentuates epithelial to mesenchymal transition in obliterative bronchiolitis. Am J Transplant.

[15] Borthwick LA, McIlroy EI, Gorowiec MR, Brodlie M, Johnson GE, Ward C, Lordan JL, Corris PA, Kirby JA, Fisher AJ (2010). Inflammation and epithelial to mesenchymal transition in lung transplant recipients: role in dysregulated epithelial wound repair. Am J Transplant.

[16] Pain M, Royer PJ, Loy J, Girardeau A, Tissot A, Lacoste P, Roux A, Reynaud-Gaubert M, Kessler R, Mussot S (2016). T cells promote bronchial epithelial cell secretion of matrix Metalloproteinase-9 via a C-C Chemokine receptor type 2 pathway: implications for chronic lung allograft dysfunction. Am J Transplant.

[17] Kawai T, Akira S (2009). The roles of TLRs, RLRs and NLRs in pathogen recognition. Int Immunol.

[18] Harada K, Sato Y, Ikeda H, Isse K, Ozaki S, Enomae M, Ohama K, Katayanagi K, Kurumaya H, Matsui A, Nakanuma Y (2009). Epithelial-mesenchymal transition induced by biliary innate immunity contributes to the sclerosing cholangiopathy of biliary atresia. J Pathol.

[19] Wu B, Crampton SP, Hughes CCW (2007). Wnt signaling induces matrix metalloproteinase expression and regulates T cell transmigration. Immunity.

[20] Rezaee F, Meednu N, Emo JA, Saatian B, Chapman TJ, Naydenov NG, De Benedetto A, Beck LA, Ivanov AI, Georas SN (2011). Polyinosinic:polycytidylic acid induces protein kinase D–dependent disassembly of apical junctions and barrier dysfunction in airway epithelial cells. J Allergy Clin Immunol.

[21] Ring L, Neth P, Weber C, Steffens S, Faussner A (2014). β-catenin-dependent pathway activation by both promiscuous “canonical” WNT3a-, and specific “noncanonical” WNT4- and WNT5a-FZD receptor combinations with strong differences in LRP5 and LRP6 dependency. Cell Signal.

[22] Parker D, Prince A (2011). Innate immunity in the respiratory epithelium. Am J Respir Cell Mol Biol.

[23] McInnes N, Davidson M, Scaife A, Miller D, Spiteri D, Engelhardt T, Semple S, Devereux G, Walsh G, Turner S (2016). Primary Paediatric bronchial airway epithelial cell in vitro responses to environmental exposures. Int J Environ Res Public Health.

[24] Ioannidis I, Ye F, McNally B, Willette M, Flaño E (2013). Toll-like receptor expression and induction of type I and type III interferons in primary airway epithelial cells. J Virol.

[25] Mayer AK, Muehmer M, Mages J, Gueinzius K, Hess C, Heeg K, Bals R, Lang R, Dalpke AH (2007). Differential recognition of TLR-dependent microbial ligands in human bronchial epithelial cells. J Immunol.

[26] Son KN, Liang Z, Lipton HL (2015). Double-stranded RNA is detected by Immunofluorescence analysis in RNA and DNA virus infections, including those by negative-stranded RNA viruses. J Virol.

[27] Negishi H, Yanai H, Nakajima A, Koshiba R, Atarashi K, Matsuda A, Matsuki K, Miki S, Doi T, Aderem A (2012). Cross-interference of RLR and TLR signaling pathways modulates antibacterial T cell responses. Nat Immunol.

[28] Nissinen L, Kahari VM (1840). Matrix metalloproteinases in inflammation. Biochim Biophys Acta.

[29] Königshoff M, Eickelberg O (2010). WNT signaling in lung disease: a failure or a regeneration signal?. Am J Respir Cell Mol Biol.

[30] Zou WW, Zou YY, Zhao ZZ, Li BB, Ran PP (2013). Nicotine-induced epithelial-mesenchymal transition via Wnt/β-catenin signaling in human airway epithelial cells. Am J Physiol Lung Cell Mol Physiol.

[31] Heijink I, Bruin HD, MVd B, Gosens R, Oosterhout AV, Postma D (2011). Role of aberrant Wnt signaling in the lung epithelial response to cigarette smoke in COPD. Eur Respir J.

[32] Reuter S, Beckert H, Taube C (2016). Take the Wnt out of the inflammatory sails: modulatory effects of Wnt in airway diseases. Lab Investig.

[33] Kim KK, Wei Y, Szekeres C, Kugler MC, Wolters PJ, Hill ML, Frank JA, Brumwell AN, Wheeler SE, Kreidberg JA, Chapman HA (2009). Epithelial cell alpha3beta1 integrin links beta-catenin and Smad signaling to promote myofibroblast formation and pulmonary fibrosis. J Clin Invest.

